# Ending AIDS as a public health threat by 2030: Time to reset targets for 2025

**DOI:** 10.1371/journal.pmed.1003649

**Published:** 2021-06-08

**Authors:** Paul R. De Lay, Adèle Benzaken, Quarraisha Abdool Karim, Sani Aliyu, Carolyn Amole, George Ayala, Kalipso Chalkidou, Judy Chang, Michaela Clayton, Aleny Couto, Carl Dieffenbach, Mark Dybul, Wafaa El Sadr, Marelize Gorgens, Daniel Low-Beer, Smail Mesbah, Jorge Saveedra, Petchsri Sirinirund, John Stover, Omar Syarif, Aditia Taslim, Safiatou Thiam, Lucy Wanjiku Njenga, Peter D. Ghys, Jose Antonio Izazola-Licea, Luisa Frescura, Erik Lamontagne, Peter Godfrey-Faussett, Christopher Fontaine, Iris Semini, Shannon Hader

**Affiliations:** 1 Independent Consultant, Washington, DC, United States of America; 2 AIDS Healthcare Foundation, Manaus, Brazil; 3 Centre for AIDS Programme of Research in South Africa (CAPRISA), Nelson Mandela School of Medicine, University of KwaZulu-Natal, Durban, South Africa; 4 Mailman School of Public Health, Columbia University, New York, United States of America; 5 Department of Microbiology, Cambridge University Hospitals, United Kingdom; 6 Clinton Health Access Initiative, Boston, Massachusetts, United States of America; 7 Alameda County Public Health Department, California, United States of America; 8 Department of Infectious Disease Epidemiology, School of Public Health, Imperial College, London, United Kingdom; 9 International Network of People Who Use Drugs (INPUD), London, United Kingdom; 10 Independent Consultant, Windhoek, Namibia; 11 STI/HIV Program at Ministry of Health, Mozambique; 12 Division of AIDS, National Institute for AIDS and Infectious Disease (NIAID), Maryland, United States of America; 13 Department of Medicine, Georgetown University, DC, United States of America; 14 ICAP, Columbia University, New York, United States of America; 15 Health, Nutrition and Population Global Practice, World Bank Group, Washington, DC, United States of America; 16 World Health Organization, Geneva, Switzerland; 17 Algiers Faculty of Medicine, People’s Democratic Republic of Algeria; 18 AHF Global Public Health Institute, University of Miami, Florida, United States of America; 19 Consultant, Bangkok, Thailand; 20 Avenir Health, Glastonbury, Connecticut, United States of America; 21 The Global Network of People Living with HIV (GNP+), Zaandam, the Netherlands; 22 Independent Consultant (previously with Rumah Cemera), West Java, Indonesia; 23 National AIDS Council, Dakar, Senegal; 24 Positive Young Women Voices, New York, United States of America; 25 Joint UN Programme on HIV/AIDS (UNAIDS), Geneva, Switzerland

## Abstract

Paul De Lay and co-authors introduce a Collection on the design of targets for ending the AIDS epidemic.

## Introduction

In 2015, the United Nations’ (UN) Sustainable Development Goal (SDG) 3 established that by 2030, the world would “end the epidemics of AIDS, tuberculosis, malaria …” [1]. As part of the SDG strategy, UNAIDS and partners developed the “Fast Track Response Strategy” in 2016 and, using standardized epidemiologic guidelines, defined “ending AIDS as a public health threat” as a 90% reduction in HIV incidence and mortality by the year 2030, compared to a baseline year of 2010 [2].

The “Fast Track Response Strategy” envisioned an accelerated ramping up of resources and programs with a set of intermediate targets for 2020, which, if achieved, would enable the attainment of the overall goals and top-line targets of 2030. There were 10 major targets and commitments established for 2020, which would result in a major reduction in HIV incidence to make the response sustainable. The Fast Track Strategy has fostered some major successes, especially around the 90–90–90 testing and treatment targets, i.e., for 90% of persons living with HIV (PLHIVs) to be aware of their status, 90% of those aware of their HIV–positive status to be on antiretroviral therapy and 90% of the latter to have achieved viral suppression. A number of countries achieved or nearly achieved these coverage levels [3]. However, overall, the global community has yet to fulfill the set of recommendations and goals of the Fast Track Strategy [4].

Over the past 5 years, funding for the global AIDS response has declined. In 2019, the global resources available for the AIDS response in low- and middle-income countries amounted to $19.8 billion, compared to the global target of $26 billion [5]. Recent data indicate that many countries were only able to reach some of their 2020 programmatic goals. The 90–90–90 testing and treatment targets have been effective globally in nearly reaching the 2020 mortality reduction target. However, mixed treatment coverage, lower emphasis on primary prevention, and insufficient tailoring of these programs to those most in need have resulted in only a 19% reduction (2019) in new HIV infections, compared to a 2020 Fast Track target of a 75% reduction [6]. Inadequate attention toward equity of access, inclusion, dealing with stigma and discrimination, social and sexual determinants of infection, achieving social justice, and implementing societal enablers, including support for community-led programs, have limited access to effective prevention, treatment, and support programs. And, finally, the world is now in the midst of the Coronavirus Disease 2019 (COVID-19) pandemic, and its implications for the public health systems and individual access to health services are evident.

Thus, in light of all of these challenges, is it still realistic to end AIDS as a public health threat by 2030 [7]? Recognizing the challenges and to ensure that we do not reverse the gains made to date, what do we need to do to get back on track to meet the UN 2030 goal to end AIDS as a public health threat?

In 2018, UNAIDS and partners initiated a new strategic planning process, which would examine progress thus far and determine where the global community has succeeded and where we are falling behind. For this process, the most current and comprehensive evidence base was elicited to identify the most effective interventions that can contribute to achieving impact over the next 5 years. This analysis was conducted in a multistakeholder, participatory process and gathered the latest effectiveness and costing data for a range of programmatic interventions. It also sought to improve the ways that mathematical modeling could be performed to translate programmatic targets into epidemiologic impact and to estimate the resources needed to achieve the new targets in low- and middle-income countries. The primary goal for this multiyear endeavor was to reexamine the range of critical targets that needed to be achieved and to assess the impact of a more comprehensive and differentiated approach to addressing the HIV epidemic that would efficiently achieve the 2030 goal. One primary output of this process was to describe a new set of interim targets for 2025, which would recalibrate the global response and assure that the 2030 goals could be achieved. We are therefore announcing a PLOS Collection in which articles will include a more detailed description of the process and present some of the conclusions.

## The process and structures

UNAIDS has produced projections of resource needs with accompanying targets 4 times since its inception in 1995. The initial projections performed in 2001 [8] and 2006 [9] focused primarily on levels of service provision and their associated cost. More recently (2011 [10] and 2016 [11]), the projections have included estimated impact of these services on incidence and mortality. This current process builds upon this previous work.

**Fig 1:**
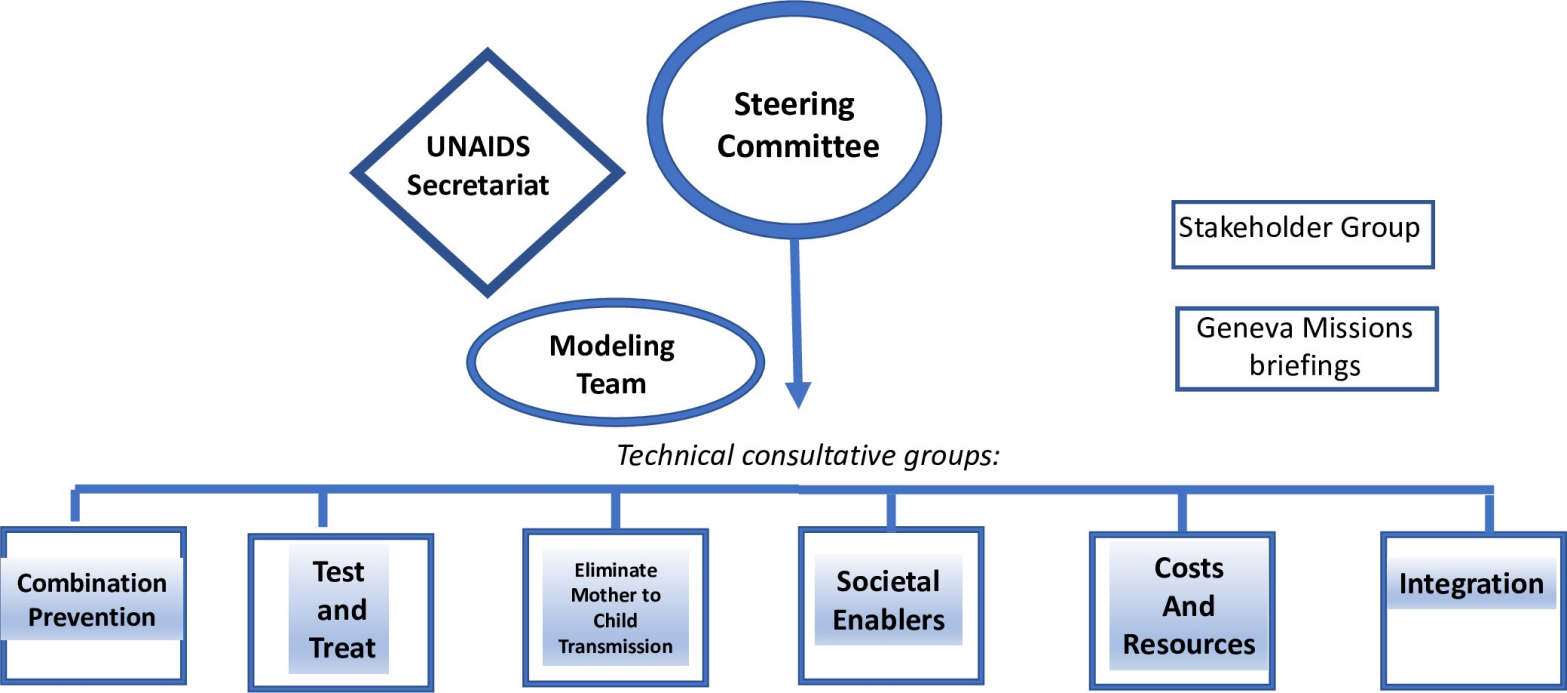
Setting AIDS 2025 targets—Functional components.

The Steering Committee, established to guide this process, initially met in July 2018, followed by 3 annual meetings. A multiyear time frame allowed for broader and more inclusive participation of critical constituencies and technical groups and provided technical groups adequate time for more extensive data gathering, building of the evidence base, and incorporation of these findings into the modeling process. A diagram of the process is provided in Fig 1.

The tasks for the Steering Committee included the framing of the exercise, the creation of technical consultative groups (TCGs), reviewing the output from these consultations, and determining how to incorporate these data into the modeling process to assess the overall programmatic strategy, the target populations, the rate of scale-up, the expected impact, and the estimated resources needed. The membership of the Steering Committee included country representatives, civil society and key population–led organizations, HIV program managers, technical experts, and relevant UN organizations.

Six TCGs were convened, each of which focused on key components of the programmatic response. These were responsible for identifying new evidence on the most effective interventions, improved methods for delivering and linking of services, relevant costs, and gathering available data on the potential impact of these programs, especially on incidence of new HIV infections and AIDS-related mortality. Participants in the TCGs included members of affected populations, program managers, researchers, service providers, strategic planners, and specialists of public health, human rights, and financial management.

A team of experts formed the Program Impact Modeling Advisory Group (PIMAG), which provided guidance and advice on modeling approaches.

The UNAIDS Secretariat provided extensive support throughout the entire process and also provided ongoing updates to a Stakeholder group consisting of primary donors and briefings of the Geneva Missions for highly impacted countries.

## Addressing the key challenges, concerns, and priorities

The articles in this Collection address the rationales for this target setting, selected inputs for key areas, the epidemiological impact modeling, and the associated resource needs estimates. These articles also describe an inventory of the most effective programmatic and social interventions, specific target populations, the evidence base for estimating program effectiveness, the lessons learned from recent trends of program scale-up, the resources needed, and the challenges presented by the concomitant COVID-19 epidemic. The Steering Committee, with guidance from the TCGs, made a number of key decisions, which would inform the inputs used in the modeling exercise.

The UN 2030 goal of ending AIDS as a public health threat should be maintained and the primary focus of the 2025 target setting be used for recalibrating the response to reach the 2030 goal and top-line targets. Given the diversity of epidemics and progress within and between countries, it was also decided to provide global-level and country-level epidemiologic impact targets, but that resource needs estimates would be limited to low- and middle-income countries.

It is important to focus on “people-centered” prevention and treatment services that reach all populations in need, using appropriate venues and approaches for delivering services, with a special focus on specific populations and subpopulations who have not been reached with traditional approaches utilized to date.

The target-setting process must build on closing the testing and treatment gaps to further enhance reductions in morbidity and mortality for all populations in need and realize the secondary prevention potential of treatment. New approaches must be used to increase treatment uptake, introduction of new drugs and drug delivery mechanisms, addressing structural and social barriers, tackling comorbidities from aging, and improvements in service delivery.

The 2 decades of experience with projection and costing models was expanded to examine the role for improving efficiencies in program implementation and how best to achieve them.

The modeling includes anticipated new technologies for prevention and treatment, including new regimens and delivery methods for treatment and preexposure prophylaxis (PrEP) that could potentially be available by 2025.

Notably, the 2025 target setting and modeling drew on the increasing body of evidence that demonstrates the critical role for actions that decrease stigma, discrimination, and criminalization of key populations and how these impact on HIV program uptake and performance and strengthen the investment case for “societal enabler” programs.

Specific attention and consideration must be placed on universal health coverage and building on how HIV response to date has supported health systems strengthening and particularly on how to optimize effectiveness and efficiencies of service integration.

A consultation of costing experts agreed on the methods for determination of the unit costs for programmatic interventions and agreed on assumptions regarding future trajectories of the costs and the benefits from achieving the targets in terms of extending lives.

Finally, it was important to understand the impact that the COVID-19 pandemic has had on the delivery of health services. UNAIDS, with multiple partners, conducted a real-time, multicountry analysis on the effect of the pandemic on various components of the HIV response, particularly examining changes in the levels of testing, uptake of treatment, and continuity of treatment.

## Conclusions

The new set of 2025 targets, including those addressing financial needs and societal enablers, has been incorporated in the UNAIDS Strategy for 2021 to 2026, which was approved by the UNAIDS Programme Coordinating Board in March 2021, and which is expected to guide and influence countries, major donors, and implementing organizations. The target-setting process has pinpointed what needs to be achieved by 2025, including the focus on people-centered and integrated services, on combination prevention, on societal enablers for the very first time, and on increased granularity to reach the SDG of ending AIDS by 2030. Achieving the targets will put the world again on track to end the AIDS epidemic as a public health threat and ensure the gains made to date are not reversed or, even worse, that the HIV/AIDS response joins the ranks of other unfinished pandemics and epidemics that the global community faces.

## Abbreviations

COVID-19: Coronavirus Disease 2019
PIMAG: Program Impact Modeling Advisory Group
PLHIV: person living with HIV
PrEP: preexposure prophylaxis
SDG: Sustainable Development Goal
TCG: technical consultative group
UN: United Nations
